# Case report: Impact of hyperthyroidism on psychotic symptoms in schizophrenia comorbid with Graves’ disease

**DOI:** 10.3389/fpsyt.2023.1219049

**Published:** 2023-07-10

**Authors:** Yukiyoshi Sumi, Sanae Kawahara, Kumiko Fujii, Mayu Yamaji, Kou Nakajima, Tsubasa Nakamura, Osamu Horikawa, Yukihiro Fujita, Yuji Ozeki

**Affiliations:** ^1^Department of Psychiatry, Shiga University of Medical Science, Otsu, Shiga, Japan; ^2^Department of Diabetology, Endocrinology and Nephrology, Shiga University of Medical Science, Otsu, Shiga, Japan

**Keywords:** schizophrenia, Graves’ disease, thyroid storm, visual hallucination, endocrinology

## Abstract

**Introduction:**

Auditory hallucinations are the most common type of hallucinations observed in schizophrenia; however, visual hallucinations are not uncommon. In Graves’ disease, depression, hypomania, and psychosis can occur. While the association between Graves’ disease and psychosis has been explored, understanding of the specific impact of thyroid dysfunction severity on psychiatric symptom severity is limited. Here, we present a case report of a patient with schizophrenia comorbid with Graves’ disease whose psychotic symptoms were impacted by hyperthyroidism.

**Case:**

The patient was a 32-year-old Japanese woman who presented with auditory and visual hallucinations, agitation, and pressured speech. The patient was diagnosed with schizophrenia comorbid with Graves’ disease and thyroid storm. The patient’s psychotic symptoms were found to be associated with fluctuations in thyroid hormone levels, and visual hallucinations were observed only during thyroid storms. Treatment involved dexamethasone, potassium iodide, bisoprolol fumarate, and methimazole for thyrotoxicosis, and a blonanserin transdermal patch, paliperidone, and paliperidone palmitate for psychotic symptoms. The patient’s auditory and visual hallucinations improved with antipsychotic treatment and decreased thyroid hormone levels.

**Conclusion:**

This case highlights the importance of monitoring thyroid function in patients with schizophrenia, particularly those with comorbid Graves’ disease. The correlation between psychiatric symptoms and thyroid hormone levels was demonstrated on an individual level over time, with symptoms worsening as thyroid hormone levels increased. Additionally, our case suggests that abnormally high thyroid hormone levels may trigger visual hallucinations in individuals with schizophrenia. Further studies are needed to elucidate the underlying mechanisms and potential treatment implications of this association.

## 1. Introduction

Schizophrenia is commonly associated with auditory hallucinations, but patients may also experience visual hallucinations over the course of their lifetime (1, 2). Graves’ disease, which is more prevalent in patients with schizophrenia than in healthy individuals (3), often presents with hyperthyroidism and psychiatric symptoms such as depression, hypomania, and psychosis (4).

Thyroid storm is a rare and life-threatening condition that can occur in individuals with Graves’ disease (5). It has a high mortality rate (8–25%) and various triggers, such as inconsistent medication use or infection. It is clinically diagnosed based on severe symptoms of hyperthyroidism. Its treatment involves a multidisciplinary approach for reducing thyroid hormone levels, managing symptoms, and addressing underlying triggers.

There exist many reports of psychosis associated with Graves’ disease and thyroid storm (6–14). Among them, the only case with coexisting psychosis spectrum disorder was that reported by Hamasaki et al. of a 76-year-old woman who had been diagnosed with schizophrenia 40 years previously (9). In terms of psychotic symptoms, all nine patients experienced delusions, three experienced auditory hallucinations (10, 11, 14), and three experienced visual hallucinations (12–14); one patient had both auditory and visual hallucinations (14), and four patients had neither auditory nor visual hallucinations (6–9). The patients experiencing visual hallucinations had a subacute clinical course, with psychosis worsening within a range of 1 week to 1 month. These patients included a patient with Graves’ disease with a 1-month history of visual hallucinations of individuals and persecutory delusions (12); a patient with Graves’ disease with a 2-month history of irritability and a 2-week history of visual hallucinations of strange figures, auditory hallucinations of strange voices, and persecutory delusions (13); and a patient with thyroid storm who had delusions, auditory hallucinations, and visual hallucinations and who rapidly worsened within 1 week (14).

Despite this, there have been no studies examining the correlation between the severity of thyroid disease and schizophrenia (15). In this report, we present a case of schizophrenia comorbid with Graves’ disease in which psychotic symptoms were affected by hyperthyroidism, with visual hallucinations occurring during a thyroid storm.

## 2. Case description

A 32-year-old Japanese woman with no history of thyroid disease presented to our hospital with auditory and visual hallucinations, agitation, and pressured speech. She had no previous clinical diagnosis of any thyroid disorder and had not undergone any thyroid-specific diagnostic evaluations. The patient had a history of delusions, offensive auditory hallucinations, and thought broadcasting since her marriage 8 years prior, and had previously presented with delusions of observation and auditory hallucinations 6 years ago. She was diagnosed with schizophrenia and prescribed perospirone 8 mg at a psychiatric clinic, but she did not have her thyroid hormone levels measured. Following treatment discontinuation and divorce 2 years prior, the patient experienced social withdrawal. Five months before presentation, she developed auditory and visual hallucinations, along with pressured speech and agitation, which prompted her father and police officers to bring her to the hospital.

Upon arrival, the patient exhibited psychomotor agitation and was found to have a blood pressure of 184/96 mmHg, a heart rate ranging from 154 to 180 bpm, and exophthalmos. Neck ultrasonography revealed an enlarged thyroid gland with increased vascularity. Laboratory examination showed elevated levels of free triiodothyronine (F-T3, >25.0 pg./mL) and free thyroxine (F-T4, >8.0 ng/dL), decreased levels of thyroid-stimulating hormone (TSH; <0.01 μU/mL), as well as elevated levels of TSH receptor antibody (>50 IU/L; reference, 0.0–1.9 IU/L), thyroid-stimulating antibody (1920 IU/mL; reference 0.0–3.2 IU/mL), and thyroglobulin antibody (867 IU/mL; reference 0.0–19.2 IU/mL). The patient was diagnosed with definite thyroid storm (16) due to probable Graves’ disease (17), as well as schizophrenia according to the Diagnostic and Statistical Manual of Mental Disorders, 5th Edition (18).

**Figure 1:**
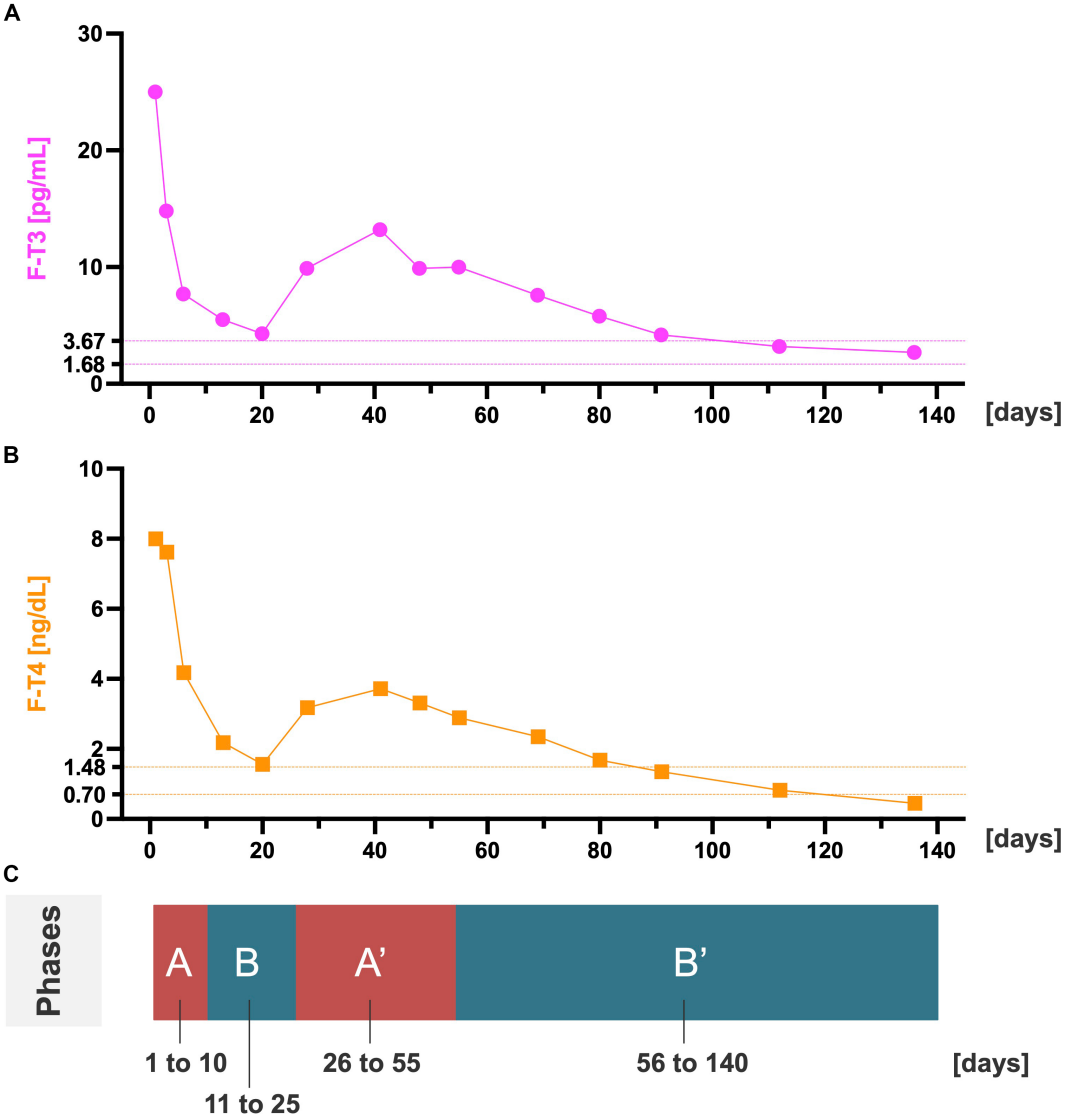
Thyroid hormone level fluctuations in the four phases. **(A)** Time series of F-T3. Dotted lines indicate the reference range for F-T3. **(B)** Time series of F-T4. Dotted lines represent the reference range for F-T4. **(C)** The four treatment phases derived from the fluctuations in thyroid hormone levels. There is an initial period of extremely high F-T3 and F-T4 levels (phase A), followed by a decline in these levels (phase B). These levels subsequently increase (phase A′) and then gradually decrease until they approach the reference range (phase B′). The thyroid-stimulating hormone levels measured simultaneously with the F-T3 and F-T4 levels were consistently below the detection sensitivity (<0.01 μU/mL). F-T3, free triiodothyronine; F-T4, free thyroxine.

**Figure 2:**
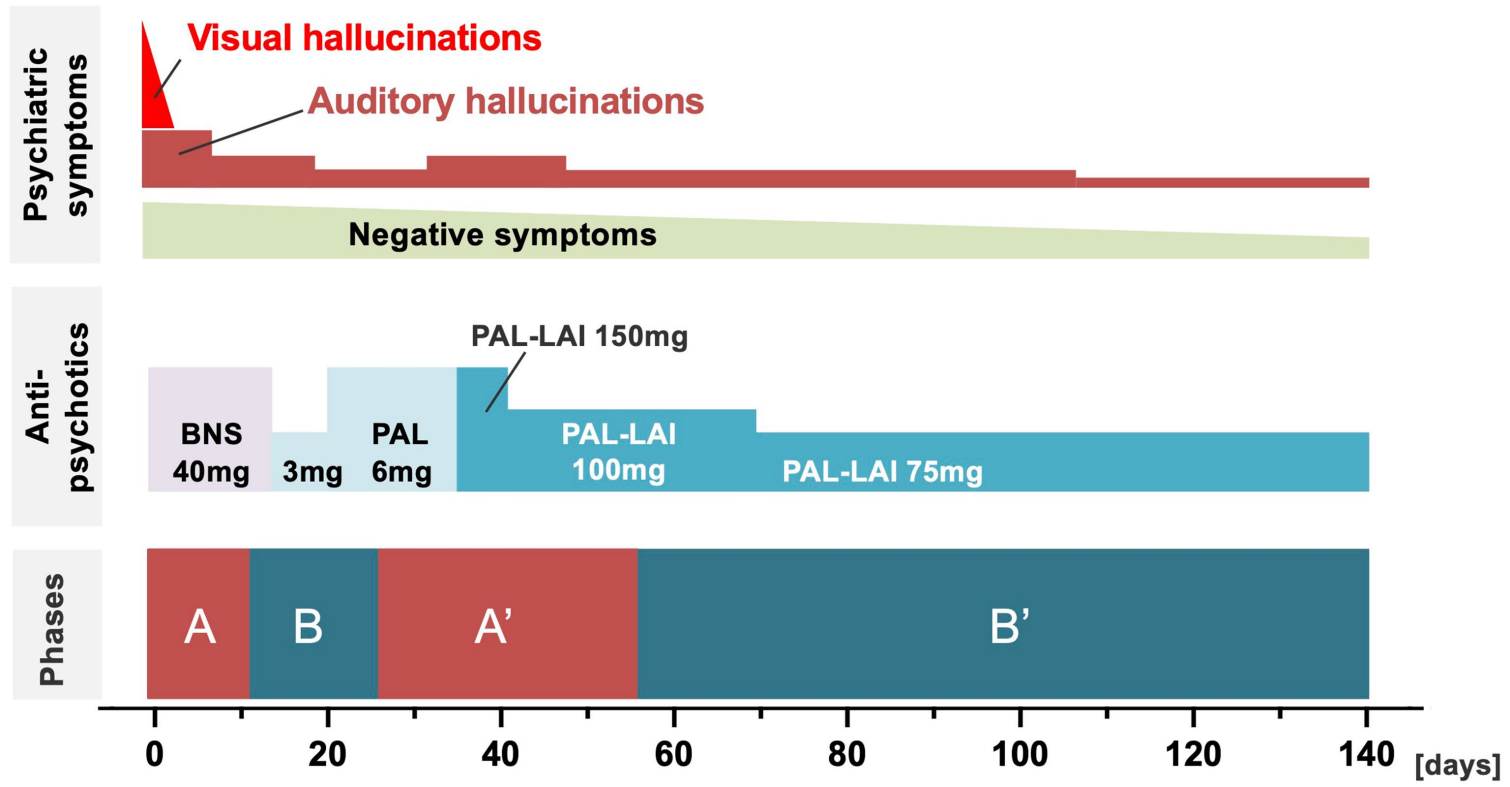
Treatment course for psychotic symptoms in the four phases. The horizontal axis indicates days of hospitalization, and three variables (psychotic symptoms, antipsychotic treatment, and phases of thyroid hormone level fluctuations) are shown. BNS, blonanserin transdermal patch; PAL, paliperidone palmitate oral route; PAL-LAI, long-acting injectable paliperidone palmitate.

To better understand the patterns of changes in F-T3 and F-T4 levels, we divided the patient’s clinical course into four phases (A, B, A′, and B′), as shown in Table 1; Figure 1. Figure 2 illustrates the patient’s treatment course, which involved hospitalization on day 1, discharge on day 130, and subsequent hospital visits on days 135 and 140. At the time of admission, the patient had extremely high levels of F-T3 and F-T4, which gradually decreased until day 20, increased again around day 40, and then decreased steadily until discharge. Throughout treatment, TSH levels remained consistently below the detection sensitivity (<0.01 μU/mL).

**Table 1:** Changes in thyroid hormones during the four treatment phases.

	Phase A	Phase B	Phase A′	Phase B′	Reference
Days of hospitalization	1 to 10	11 to 25	26 to 55	56 to 140	
Free triiodothyronine (F-T3) (pg/mL)	15.8 ± 7.1	4.9 ± 0.6	10.8 ± 1.4	4.7 ± 1.8	1.68–3.67
Free thyroxine (F-T4) (ng/dL)	6.60 ± 1.72	1.87 ± 0.31	3.28 ± 0.30	1.33 ± 0.66	0.70–1.48
Thyroid-stimulating hormone (TSH) (μU/mL)	<0.01	<0.01	<0.01	<0.01	0.35–4.94

The table shows thyroid hormone levels in phases A, B, A′, and B′ (mean ± standard deviation).

**Figure 3:**
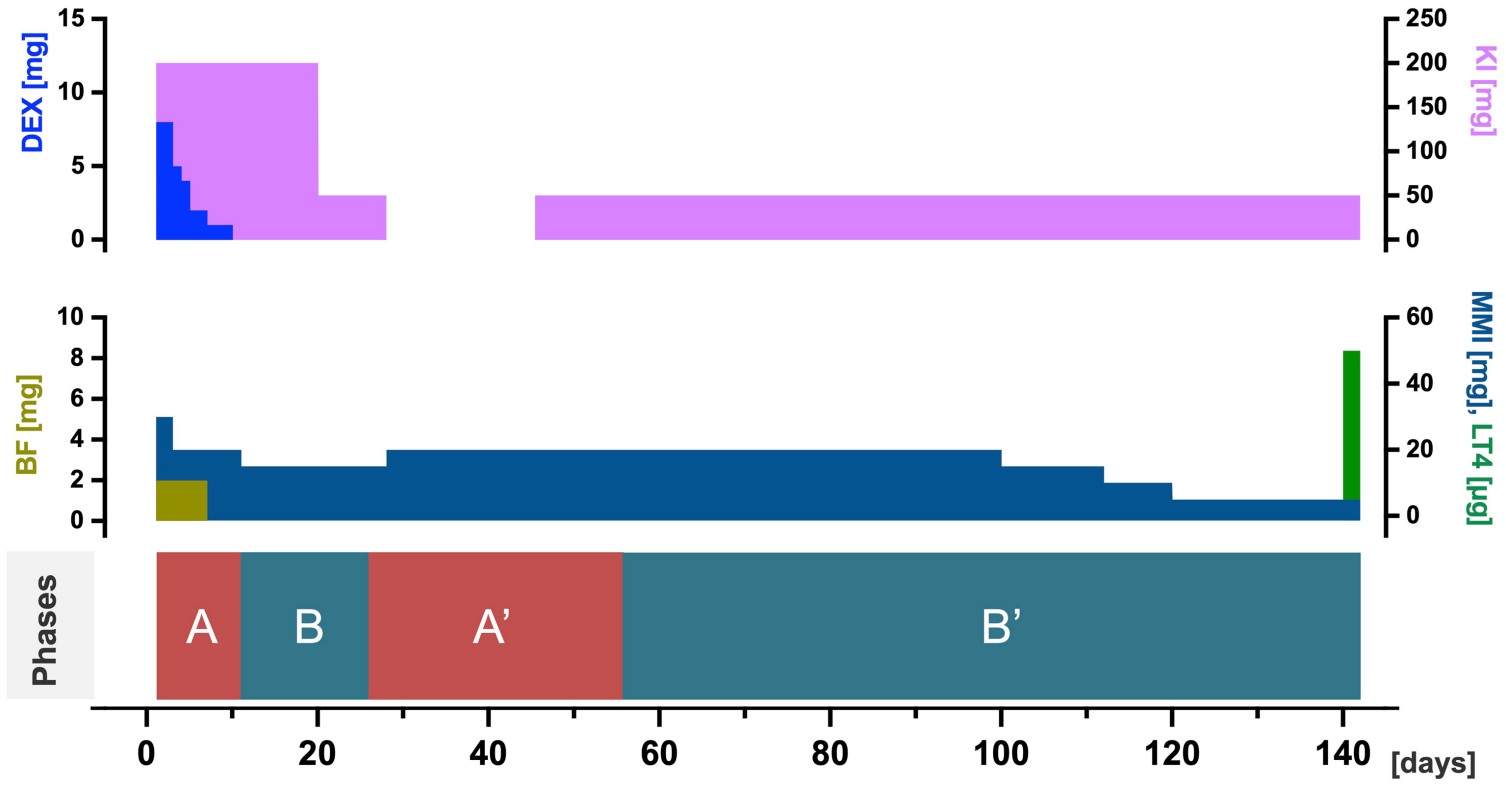
Treatment course for hyperthyroidism in the four phases. The horizontal axis indicates days of hospitalization, and the three variables (treatment with DEX and KI; BF, MMI, and LT4; and phases of thyroid hormone level fluctuations) are shown. BF, bisoprolol fumarate; DEX, dexamethasone; KI, potassium iodide; LT4, levothyroxine; MMI, thiamazole.

During phase A, for managing thyrotoxicosis, the patient received treatment with 8 mg of dexamethasone, 200 mg of potassium iodide, 2 mg of bisoprolol fumarate, and 30 mg of methimazole, while her psychotic symptoms were treated with a 40 mg/day blonanserin transdermal patch (details are shown in Figures 2, 3). The patient’s auditory/visual hallucinations, agitation, and pressured speech disappeared within 2 days. However, she continued to talk to herself actively and reported hearing voices from her “friends,” indicating persisting auditory hallucinations. The doses of the medications for treating hyperthyroidism were gradually reduced according to the clinical manifestations and thyroid hormone levels. Specifically, the dexamethasone dose was tapered from an initial dose of 8 mg, and dexamethasone was discontinued on day 10. The methimazole dose was tapered to a daily dose of 20 mg from day 3 onwards. Bisoprolol fumarate was discontinued on day 7.

In phase B, F-T3 and F-T4 levels decreased, as did the auditory hallucinations and self-talk. The patient’s vital signs mostly remained within the normal range. For example, on day 13, the blood pressure was 130/70 mmHg and pulse rate ranged from 65 to 77 bpm. The patient’s treatment was adjusted considering the potential future administration of long-acting injectable paliperidone palmitate (PAL-LAI). From day 13 onward, the use of the blonanserin transdermal patch was discontinued, and oral administration of 3 mg of paliperidone was started. On day 20, the paliperidone dose was increased to 6 mg. For managing hyperthyroidism, the methimazole dose was tapered to 15 mg on day 11. Additionally, the potassium iodide dose was tapered to 50 mg on day 20. By day 23, her Positive and Negative Syndrome Scale (PANSS) (19) score was 87 (positive: 19; negative: 27; global psychopathology: 41).

During phase A′, F-T3 and F-T4 levels increased considerably, and auditory hallucinations and self-talk increased again, but no visual hallucinations were observed. Among the medications for treating psychiatric symptoms, on day 34, oral paliperidone administration was discontinued and 150 mg PAL-LAI administration was initiated. The PAL-LAI dose was tapered to 100 mg on day 41. For treating hyperthyroidism, the methimazole dose was increased to 20 mg on day 28. Potassium iodide administration was initially discontinued, but it was restarted at a dose of 50 mg on day 45 due to an increasing trend in F-T3 and F-T4 levels.

In phase B′, F-T3 and F-T4 levels again decreased. Additionally, the patient’s psychiatric symptoms gradually improved. Notably, logorrhea disappeared, speech coherence improved, and the patient showed improved engagement in interactions with healthcare providers and other hospitalized patients and participation in group therapy. She experienced fewer auditory hallucinations, expressed discomfort with them, and gradually gained insight into her psychosis. The patient continued PAL-LAI treatment according to the prescribed regimen, receiving injections of 75 mg on days 69, 97, and 125. For treating hyperthyroidism, the potassium iodide dose was maintained at 50 mg, while the methimazole dose was reduced to 15 mg on day 100, 10 mg on day 112, and 5 mg on day 120. On day 140, 50 mg levothyroxine administration was initiated. By day 125, her PANSS score had decreased to 55 (positive: 12; negative: 17; global psychopathology: 26), and she was discharged on day 130. The treatment course is shown in Figures 2, 3.

## 3. Discussion

We reported a case of schizophrenia comorbid with Graves’ disease with two unique characteristics: 1) psychotic symptoms changed as thyroid hormone levels fluctuated and 2) visual hallucinations appeared only when abnormally high thyroid hormone levels were present.

### 3.1. Relationship between psychiatric symptoms and thyroid hormone levels

Free hormones, which do not bind to transport proteins, exhibit physiological activity (20). Furthermore, a correlation between elevated serum thyroid hormone levels and psychiatric symptom exacerbation, which can lead to hospitalization in patients with thyroid disorders, has been reported (21). Thus, heightened serum thyroid hormone levels may have an important influence on psychiatric symptoms in individuals with Graves’ disease.

In our case, we demonstrated the relationship between thyroid hormone levels and the severity of psychiatric symptoms on an individual level through time series analysis. One-way analysis of variance of F-T3 and F-T4 levels in the four phases showed significant differences between groups for both F-T3 and F-T4 levels (F-T3, F (3, 10) = 5.263, *p* = 0.020; F-T4, F (3, 10) = 15.962, *p* < 0.001). In addition, post-hoc analysis with Tukey’s multiple comparison showed that the F-T3 level was higher in phase A than in phase B′ and the F-T4 level was higher in phase A than in phases B, A′, and B′ (Table 2). Statistical analysis was performed using JASP version 0.17.1 (JASP Team, Amsterdam, the Netherlands). Psychiatric symptoms tended to worsen with increasing thyroid hormone levels, with psychosis worsening more during phases A/A′ than in phases B/B′, suggesting a link between increased thyroid hormone levels and severe psychiatric symptoms. Prior reports have documented the relationships between psychiatric symptoms and exacerbations of thyroid disease (4, 22). However, to the best of our knowledge, this is the first report on the changes in psychiatric symptoms in relation to thyroid hormone levels in a patient with thyroid disease comorbid with schizophrenia.

**Table 2:** Multiple group comparisons of thyroid hormone levels using one-way ANOVA.

	Source	Sum of squares	df	Mean square	*F*	*p*
Free triiodothyronine (F-T3)	Between groups	278.058	3	92.686	5.263	**0.020**
	Within groups	176.097	10	17.61		
Free thyroxine (F-T4)	Between groups	55.562	3	18.521	15.962	**<0.001**
	Within groups	11.603	10	1.16		

Post-hoc analysis	Comparison	*p*
Free triiodothyronine (F-T3)	Phase A – Phase B	0.069
	Phase A – Phase A′	0.428
	Phase A – Phase B′	**0.020**
	Phase B – Phase A′	0.416
	Phase B – Phase B′	1.000
	Phase A′ – Phase B′	0.203
Free thyroxine (F-T4)	Phase A – Phase B	**0.003**
	Phase A – Phase A′	**0.011**
	Phase A – Phase B′	**< 0.001**
	Phase B – Phase A′	0.469
	Phase B – Phase B′	0.930
	Phase A′ – Phase B′	0.089

Upper half of the table shows the results of the one-way ANOVA for free triiodothyronine (F-T3) and free thyroxine (F-T4) in the four phases, showing significant differences between groups for both F-T3 and F-T4.

Lower half of the table shows the post hoc analysis with Tukey’s multiple comparison: Phase A > Phase B′ for F-T3 and Phase A > Phase B, A′, B′ for F-T4.

Bold text represents statistical significance (*p*< 0.05). ANOVA, analysis of variance.

Immune dysregulation and physical/psychological stress are considered to impact psychiatric symptoms in patients with autoimmune diseases (15). In this case, tachycardia and emotional changes, commonly seen in thyroid disease (5), may have acted as physical/psychological stressors and contributed to the exacerbation of psychiatric symptoms.

Treatment for thyroid disease likely played a role in the rapid improvement of the patient’s psychiatric symptoms after admission. The patient received a blonanserin transdermal patch, dexamethasone, thiamazole, potassium iodide, and bisoprolol fumarate to treat psychosis and hyperthyroidism (Figure 3). Dexamethasone, which reduces the conversion of thyroxine to triiodothyronine (5), was prescribed from days 1 to 9 and may have contributed to the quick improvement in psychiatric symptoms during the first 2 days of hospitalization. Thiamazole inhibits thyroid hormone production and has immunosuppressive effects (23, 24), and potassium iodide decreases thyroid hormone release and vascularity of the thyroid gland (25). Bisoprolol may have also played a role in reducing tachycardia and aggression and contributed to the amelioration of psychosis (26).

Although the patient’s psychiatric symptoms improved from phase A to B, they worsened again in phase A’. Several factors may have contributed to this, such as increased production of thyroid hormones after discontinuing potassium iodide (25) on day 27 and delayed increase in paliperidone serum concentration following PAL-LAI introduction on day 34 (27). Psychiatric symptoms stabilized after administering 100 mg of PAL-LAI on day 41 and 75 mg of PAL-LAI on day 69.

The patient’s history reveals a lack of previous identification or diagnostic evaluation for thyroid disorders. Six years ago, she was diagnosed with schizophrenia based on a history of persistent delusions, auditory hallucinations, and thought broadcasting. However, the extent of thyroid dysfunction was not assessed at the time of schizophrenia diagnosis. Therefore, definitively determining whether the previous psychosis episode was primarily attributable to schizophrenia or Graves’ disease is challenging. Nonetheless, notable differences in symptoms exist between the episode from 8 years ago and the current episode, which commenced 5 months ago. While the previous episode was characterized by auditory hallucinations, paranoid delusions, and social withdrawal, the current episode was marked by agitation, visual hallucinations, and pressured speech. Considering the distinctive patterns of psychiatric symptoms manifested, we can posit that the current episode may have been influenced by a novel factor, potentially thyroid disease.

Basic research studies have demonstrated that F-T3 may activate the limbic system via catecholamines and that prolonged type 2 iodothyronine deiodinase activity in the central nervous system may prolong psychiatric symptoms (28, 29). However, the relationship between hyperthyroidism and psychiatric symptoms in clinical settings remains poorly understood. To gain a better understanding of this relationship, future research should investigate the association between thyroid hormone levels and the severity of psychotic symptoms, as demonstrated in this case. Additionally, further studies could explore the potential of using thyroid hormone levels as a biomarker for the severity of psychotic symptoms. Such investigations could assist clinicians in managing comorbid schizophrenia and thyroid diseases.

### 3.2. Visual hallucinations

Visual hallucinations are a well-known symptom of organic disorders such as neurodegenerative and eye diseases, but they are also observed in schizophrenia (1, 2). The prevalence of auditory hallucinations in schizophrenia is high (59%), while that of visual hallucinations is 27% (2). Brain imaging studies have shown increased activity in the visual association cortex and primary visual cortex in patients with psychosis who have visual hallucinations (30), and dysconnectivity in the temporal and occipital cortices has been implicated as a potential mechanism (31). Additionally, dysfunction in attentional and executive/top-down mechanisms is believed to be a fundamental pathology of visual hallucinations in patients with psychosis, similar to that in patients with neurodegenerative diseases (2). Social, personal, and psychological factors have also been shown to influence the development of visual hallucinations in patients with psychosis (32).

Our case suggests that endocrine abnormalities may play a role in the occurrence of visual hallucinations in patients with schizophrenia. While the patient had experienced auditory hallucinations for 8 years, visual hallucinations only appeared 5 months before and a few days after her hospitalization for her 8-year history of schizophrenia. Several months before admission and during the early phase of her hospitalization, she experienced a thyroid storm. The onset of visual hallucinations coincided with the thyroid storm, which suggests that abnormally high thyroid hormone levels induced a different type of hallucination. Further research is needed to investigate the relationship between visual hallucinations and endocrine abnormalities, as well as brain imaging and electrophysiological findings. Investigating the prevalence and incidence of visual hallucinations in patients with Graves’ disease and schizophrenia, separately and in comorbidity, could provide valuable insight into the underlying mechanisms and potential risk factors for these symptoms.

## 4. Conclusion

This case report highlights a potential correlation between worsening thyroid disease and exacerbation of psychotic symptoms in a patient. Additionally, it suggests that a thyroid storm may contribute to visual hallucinations in patients with schizophrenia. Clinicians should be aware of the possible impact of thyroid disease exacerbation on psychiatric symptoms and carefully monitor for changes in their quality or severity.

## Data availability statement

The original contributions presented in the study are included in the article/supplementary material, further inquiries can be directed to the corresponding author.

## Ethics statement

Ethical review and approval was not required for the study on human participants in accordance with the local legislation and institutional requirements. The patients/participants provided their written informed consent to participate in this study. Written informed consent was obtained from the individual(s) for the publication of any potentially identifiable images or data included in this article.

## Author contributions

YS and SK: conception and design of the study. YS, SK, MY, and TN: acquisition and analysis of data. YS, SK, KF, and YO: drafting the manuscript or figures. YS, SK, KF, MY, KN, TN, OH, YF, and YO: review and critique. All authors contributed to the article and approved the submitted version.

## Funding

This study was supported by the JSPS KAKENHI (grant number 21K15745).

## Conflict of interest

The authors declare that the research was conducted in the absence of any commercial or financial relationships that could be construed as a potential conflict of interest.

## Publisher’s note

All claims expressed in this article are solely those of the authors and do not necessarily represent those of their affiliated organizations, or those of the publisher, the editors and the reviewers. Any product that may be evaluated in this article, or claim that may be made by its manufacturer, is not guaranteed or endorsed by the publisher.
